# Micro-Spherical BiOI Photocatalysts for Efficient Degradation of Residual Xanthate and Gaseous Nitric Oxide

**DOI:** 10.3390/nano14070576

**Published:** 2024-03-26

**Authors:** Qianqian Nie, Liuhu Jia, Guoqing Zhang, Jiewei Xie, Jiayou Liu

**Affiliations:** 1 Key Laboratory of Coal Processing and Efficient Utilization, Ministry of Education, Xuzhou 221116, China; cumtnqq@hotmail.com (Q.N.); 18303607206@163.com (L.J.); guoqingzhang@cumt.edu.cn (G.Z.); 17852030785@163.com (J.X.); 2 School of Chemical Engineering and Technology, China University of Mining and Technology, Xuzhou 221116, China

**Keywords:** BiOI, photocatalysis, visible light, isobutyl sodium xanthate, NO

## Abstract

BiOI microspheres were synthesized using the solvothermal method for the degradation of residual xanthate and gaseous nitric oxide (NO) under visible light irradiation. The as-prepared BiOI nanomaterials were then characterized using various technologies, including XRD, FE-SEM, TEM, UV-Vis DRS, and XPS. The photodegradation results show that the removal efficiency of isobutyl sodium xanthate can reach 98.08% at an initial xanthate concentration of 120 mg/L; that of NO is as high as 96.36% at an inlet NO concentration of 11 ppm. Moreover, the effects of operational parameters such as catalyst dosage, initial xanthate concentration, and pH value of wastewater on the removal of xanthate were investigated. The results of scavenging tests and full-spectrum scanning indicate that ·O_2_^−^ radicals are the main active species in xanthate degradation, and peroxide xanthate is an intermediate. The reusability of BiOI was explored through cyclic experiments. Furthermore, the reaction path and the mechanism of NO removal using BiOI were analyzed, and the main active species was also ·O_2_^−^. It is concluded that BiOI photocatalysts have high potential for wastewater treatment and waste gas clean-up in the mineral industry.

## 1. Introduction

The rapid development of society relies heavily on mineral industries. However, the increasing pollution related to mineral processing and smelting has become a considerable challenge to the ecosystem [1]. Flotation wastewater and smelting waste gas are among the most serious environmental issues that demand effective treatment in the mineral industry.

In a flotation process, a large amount of wastewater containing residual reagents is discharged, causing severe water pollution [2]. Xanthates, one of the most commonly used collectors in the flotation of sulfide minerals, can harm the livers, nervous systems, and hematopoietic systems of aquatic organisms and human beings [3,4]. In addition, the residual xanthate in the reused water has a negative impact on the flotation [5]. Therefore, the residual xanthate in flotation wastewater needs to be well treated before discharging or recycling. Many traditional methods, including the Fenton-like method [6], biodegradation [7], and physical adsorption [8], have been developed to remove xanthates. However, they still have disadvantages such as high cost, secondary pollution, and a long degradation period.

Air pollution is another serious environmental issue in mineral engineering. Nitric oxide produced during the sintering process is difficult to control [9,10], and it is the main culprit of acid rain, photochemical smog, and ozone layer depletion, which threatens the environment and human safety [11,12]. Although the selective catalytic reduction (SCR) method [13,14] can achieve efficient denitrification, its catalyst deactivation, high temperature requirements, and expensive reducing agents boost operating costs. Other existing technologies also have disadvantages [15,16] and are not commonly accepted in real applications. It is, therefore, desired to develop alternatives to NO reduction.

Photocatalysis has been widely used as a cost-effective and green technology in environmental remediation in recent years [17,18,19,20,21,22,23]. It converts sustainable solar energy into chemical energy to efficiently degrade pollutants. However, traditional photocatalysts like TiO_2_ [24] and ZnO [25] have wide band gaps of around 3.2 eV. They only respond to ultraviolet light, which only accounts for 4% of solar energy [26], while visible light constitutes 44% of solar energy but cannot be effectively utilized. Hence, visible light-responsive photocatalysts have gradually become mainstream in photocatalytic studies.

BiOI is an n-type semiconductor with a narrow band gap [27,28]. It is widely used in visible light driven photocatalysis due to its non-toxicity, strong chemical stability, and high catalytic efficiency. Chen et al. [29] synthesized BiOI microspheres using the solvothermal method for the degradation of methylene orange (MO). They stated that 40% MO could be degraded under visible light irradiation during a 50 min period. Bai et al. [30] used BiOI to achieve removal efficiencies of 100%, 72%, and 83% for rhodamine B (RhB), phenol, and bisphenol A (BPA), respectively. They believed that ·O_2_^−^ and ·OH species played main roles simultaneously. However, as far as we know, the degradation of xanthate using BiOI has not been reported.

Moreover, there have been some reports on the degradation of NO using BiOI. However, the explanations of previous studies about the main active species in NO photodegradation are inconsistent. Dong et al. [31] reported that ·OH was the primary reactive species for NO removal. On the contrary, Zhu et al. [32] believed that the main active species was ·O_2_^−^ rather than ·OH. Therefore, it is necessary to revisit the contradiction in the literature to gain a better understanding of the mechanism behind NO reduction using BiOI.

In this paper, BiOI prepared using the solvothermal method was used to degrade xanthate wastewater and gaseous NO. First, the synthesized BiOI was characterized using various technologies, including XRD, FE-SEM, TEM, UV-Vis DRS, and XPS, to investigate its crystal phase, morphology, surface element, and optical properties. Then, the effects of catalyst dosage, xanthate concentration, pH value, and the concentrations of Ca^2+^ and Mg^2+^ ions on the removal of xanthate were explored. In addition, the reaction path and mechanism of photodegradation of xanthate were discussed based on scavenging tests, chemical oxygen demand (COD) date, and full-spectrum scanning. Moreover, NO removal was carried out under visible light irradiation. Its reaction path was analyzed according to detected products, and the main active species in NO removal were determined by isolating oxygen and water tests, which can inhibit the production of ·O_2_^−^ and ·OH, respectively.

## 2. Experimental Section

### 2.1. Materials and Reagents

The reagents used in this study include bismuth nitrate pentahydrate (Bi(NO_3_)_3_·5H_2_O), potassium iodide (KI), sodium hydroxide (NaOH), magnesium chloride hexahydrate (MgCl_2_·5H_2_O), calcium chloride dihydrate (CaCl_2_·2H_2_O), nitric acid (HNO_3_), ethylene glycol, ether absolute, tert-butanol (TBA), p-benzoquinone (BQ), acetone, anhydrous ethanol, and methanol (MeOH). They were purchased from Sinopharm Chemical Reagent Co., Ltd. (Shanghai, China). They were of analytical grade and used directly without further purification. Isobutyl sodium xanthate with industrial purity was purchased from Qingdao Liantuo Chemical Co., Ltd. (Qingdao, China). Ultrapure water with a resistivity of 18.2 MΩ⋅cm^−1^ used in the experiment was produced by Direct-Q 5 UV System (Merck Millipore, Darmstadt, Germany).

### 2.2. Preparation of Photocatalyst BiOI

BiOI samples were prepared using a solvothermal method according to a previous report [33]. First, Bi(NO_3_)_3_·5H_2_O (4 mmol) and KI (4 mmol) were dissolved separately in 35 mL glycol solution and stirred at room temperature for 30 min. A mixture was then produced by adding the KI solution slowly to the Bi(NO_3_)_3_·5H_2_O solution. The mixture was stirred at room temperature for another 30 min to form a precursor solution. Afterwards, the precursor was poured into a 100 mL autoclave made of Teflon-lined stainless steel and heated at 160 °C for 12 h. After cooling to room temperature, the solids were separated using a centrifuge at 4500 RPM. In order to remove ion and solvent impurities, the products were washed with ultrapure water and anhydrous ethanol three times. Finally, the resultant solids were dried at 60 °C for 12 h for subsequent applications.

### 2.3. Characterization Methods

The prepared catalysts were characterized using various methods. The crystal phases and structures of all the samples were analyzed via X-ray diffraction (XRD, Bruker D8-Advance, Germany, 40 kV, 30 mA Cu- κβ radiation, λ = 1.5418 Å). X-ray photoelectron spectroscopy with an Al Kα X-ray radiation source (XPS, Thermo Scientific K-Alpha, Waltham, MA, USA) was used to analyze the chemical composition. The morphological structure was determined using a field emission scanning electron microscope (FE-SEM, Hitachi-SU8220, USA) and a transmission electron microscope (TEM, Tecnai G2-F20 FEI, USA). The lattice spacing of BiOI was measured with a high-resolution transmission electron microscope (HRTEM, Tecnai G2-F20 FEI, USA). Elements were analyzed using an energy-dispersive spectrometer (EDS, Bruker Nano GmbH-XF lash Detector, Germany). A UV-Vis diffused reflectance spectrometer (UV-Vis DRS, Hitachi U-3900H, Japan) was employed to measure optical properties. The specific surface area and pore structure measurements were performed using the Brunauer–Emmett–Teller (BET) method (ASAP 2460 Version 3.01, USA). Mott–Schottky plots were acquired in an electrochemical workstation (CHI660E, China); 0.5 M Na_2_SO_4_ was chosen as the electrolyte. The COD was determined using a COD analyzer (Huatong Huanbao CTL-12, China).

### 2.4. Purification of Isobutyl Sodium Xanthate

The isobutyl sodium xanthate which was purchased was of industrial grade; therefore, it was purified to remove unknown impurities. The specific purification method can be found in our previous report [34], and is briefly summarized as follows: 2 g of xanthate was thoroughly ground before it was dissolved in 5 mL of acetone solution. A yellow solution was obtained after filtration. Anhydrous ether was added to the solution until no solid was further precipitated. The purified xanthate, which consisted of faint yellow solids, was then produced after another filtration. The purified xanthate was placed into a vacuum oven and dried at 40 °C for 6 h. After drying, xanthate was put into a brown reagent bottle and stored in a desiccator.

### 2.5. Photocatalytic Degradation Experiments

#### 2.5.1. Photocatalytic Degradation of Xanthate

Photocatalytic degradation of xanthate was conducted in a photoreactor equipped with a 300 W Xe lamp; a 380 nm filter was used to remove UV lights. The light intensity, measured by a radiometer (FZ-A, Beijing Normal University), was set as 31.45 mW/cm^2^. The initial volume of wastewater for treatment was maintained at 50 mL. First, two blank tests were conducted under irradiation without a catalyst and under a dark environment with a catalyst to show the effectiveness of photocatalysis at the concentration of xanthate of 120 mg/L. The xanthate concentration ranged from 60 to 160 mg/L, and the BiOI catalyst dosage was between 0.1 and 0.5 g/L. The pH of xanthate wastewater was regulated to 4.490, 6.814, 8.044 (original solution), 9.775, and 10.225 with 0.1 M NaOH and/or HNO_3_ solution. The concentrations of Ca^2+^ and Mg^2+^ were maintained at 20–30 mg/L with CaCl_2_ and MgCl_2_ solutions, respectively.

For a typical test, 50 mL of wastewater was first stirred under dark conditions for 45 min to achieve equilibrium between adsorption and desorption before light was turned on to start photodegradation. The photocatalytic treatment lasted for 120 min; 1.5–2.0 mL of the solution was sampled every 15 min. Afterwards, the collected sample was centrifuged and the supernatant was analyzed using the UV-Vis spectrophotometer. Moreover, the stability of the BiOI was evaluated via cyclic tests. After each photodegradation cycle, the photocatalyst was recovered by means of centrifugation and then washed with ultrapure water in an ultrasonic bath. The cleaned photocatalysts were then dried at 60 °C for next cycle.

In order to study the role of the active species during the photodegradation of xanthate, methanol (MeOH), tert-butanol (TAB), and p-benzoquinone (BQ) were added into 50 mL xanthate wastewater to capture photogenerated electrons (h^+^), hydroxyl radicals (·OH), and superoxide radicals (·O_2_^−^), respectively [35,36].

#### 2.5.2. Photocatalytic Degradation of NO

Photocatalytic degradation of NO was carried out in a rectangular ISO reactor [11] with a volume of 25 cm^3^ (10 × 5 × 0.5 cm^3^). The reactor was covered with a piece of heat-resistant quartz glass (6 × 11 cm^2^) that allowed simulated sunlight to penetrate. Simulated sunlight was provided by a 300 W Xe lamp (380 nm cut-off filter) with a light intensity of 59.55 mW/cm^2^. The Xe lamp was placed 22 cm above the quartz glass. For each photocatalytic reaction, 50 mg BiOI was loaded onto the glass slide (5 × 10 cm^2^). The concentration of NO was regulated by mass flow controllers and was measured using a continuous FTIR analyzer (MKS MultiGas 2060). The simulated flue gas containing NO (11 ppm), O_2_ (5%), N_2_, and H_2_O (50–70% RH) was used in the NO photooxidation.

## 3. Results and Discussion

### 3.1. Characterization of Samples

#### 3.1.1. XRD Results

**Figure 1:**
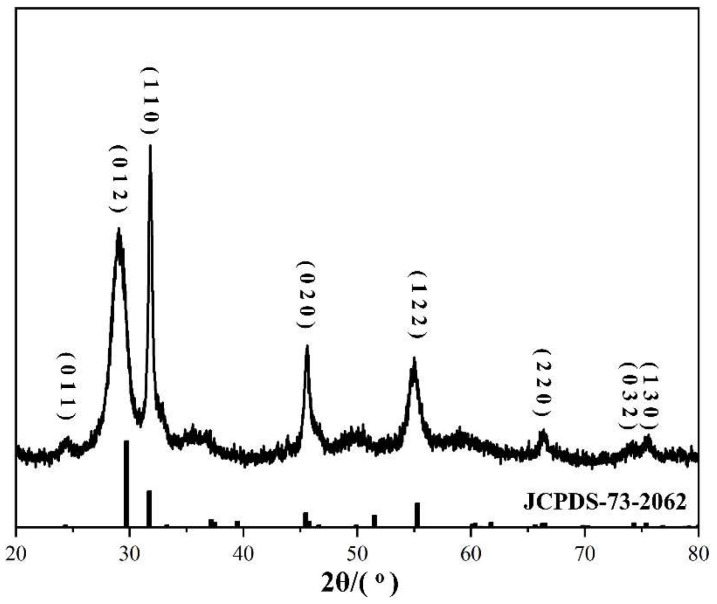
XRD patterns of the synthesized BiOI nanomaterials.

The phase structure of the BiOI sample was detected by X-ray diffraction (XRD) characterization. As shown in Figure 1, the diffraction peaks at 29.74°, 31.74°, 45.49°, 55.30°, 66.30°, 74.32°, and 75.37° were assigned to the (012), (110), (020), (122), (220), (032), and (130) planes of pure BiOI, respectively, with space group P4/nmm (JCPDS No. 73-2062). Furthermore, the sharp diffraction peaks indicate the strong crystallinity of the synthesized samples. No other characteristic peaks were identified, indicating that the purity of BiOI was high.

#### 3.1.2. Morphology and Microstructure

**Figure 2:**
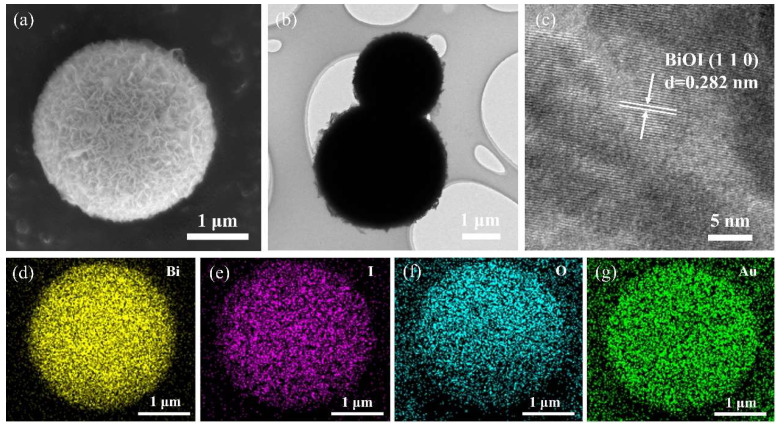
(**a**) FE-SEM, (**b**) TEM, and (**c**) HR-TEM images of BiOI; EDS mapping of (**d**) Bi, (**e**) I, (**f**) O, and (**g**) Au elements.

Figure 2 shows the morphology of BiOI, determined using FE-SEM and TEM techniques. Regular BiOI microspheres with sizes ranging from 2 to 3 μm can be observed in Figure 2a. The microsphere is comprised of numerous curved nanosheets, which favors contact with pollutant molecules and provides numerous active sites for reaction. Figure 2b shows a TEM image of the BiOI microsphere with a fluffy edge, which corresponds to the nanosheets on the surface of BiOI. Furthermore, these nanosheets were further characterized via HR-TEM to identify the lattice. In Figure 2c, clear lattice fringes with 0.284 nm of spacing can be observed, corresponding to the (110) plane of BiOI.

Figure 2d–g show the EDS mappings of BiOI microspheres. Bi, I, O, and Au elements can be detected, and their mapping images also appear in a spherical shape. The Bi, I, and O elements were derived from BiOI, whereas the detected Au was introduced during sample preparation.

**Figure 3:**
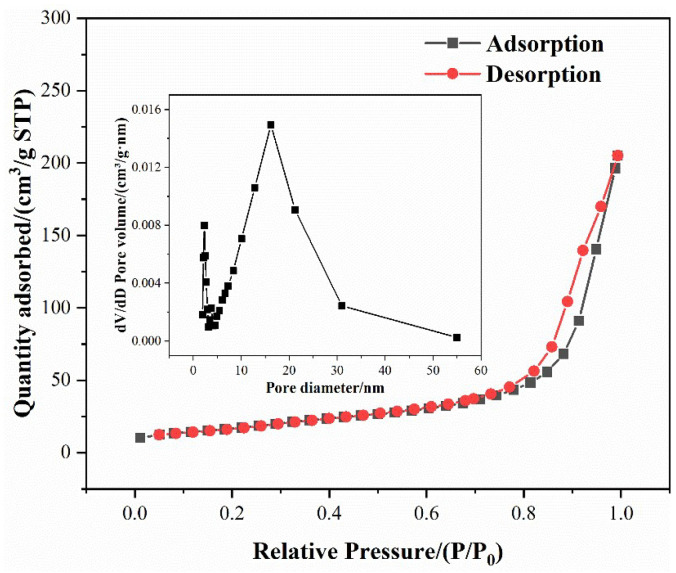
Nitrogen adsorption–desorption isotherm and the corresponding BJH pore size distribution curve (inset) of BiOI.

Figure 3 shows the N_2_ adsorption–desorption isotherm of BiOI. The isotherm is a type IV curve with an H3 hysteresis loop under relative pressures ranging from 0.7 to 1.0, which indicates the presence of interstitial mesopores with slit-like shapes formed by aggregated nanosheets [37]. Moreover, the BET surface area of BiOI was 61.53 m^2^/g. The corresponding pore size was distributed from 2 to 55 nm, as demonstrated in the inset of Figure 3. The most probable aperture of BiOI was 16.18 nm, which may correspond to the slit-like mesopores. In addition, some micropores 2.29 nm in diameter were present. This large specific surface and rich pore structure of BiOI provided a large number of adsorption sites for pollutants in the photocatalytic reaction.

#### 3.1.3. XPS Analysis

**Figure 4:**
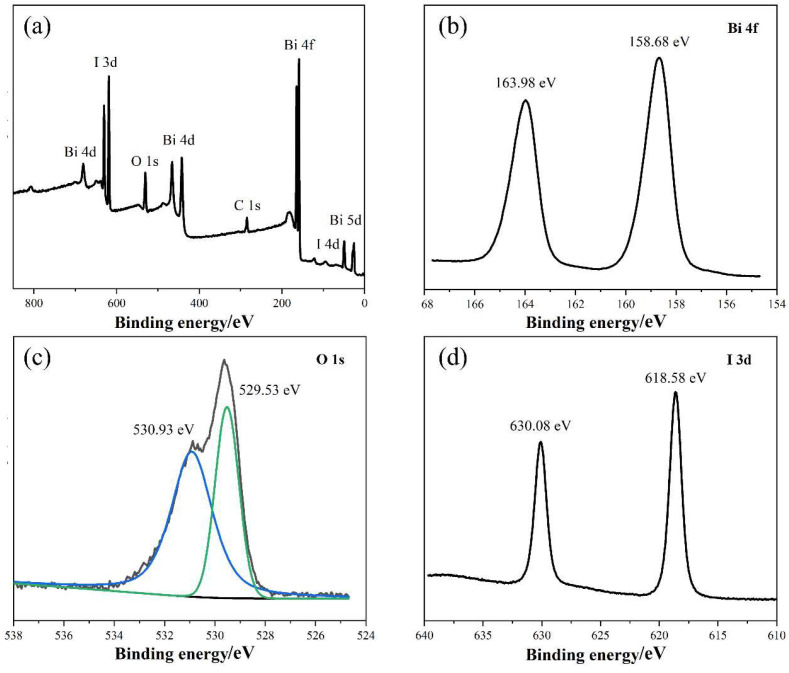
X-ray photoelectron spectroscopy analysis of BiOI: (**a**) full-spectrum scan, and high-resolution XPS spectra of (**b**) Bi, (**c**) O, and (**d**) I elements.

The surface chemical states and constituent elements of BiOI were analyzed using XPS measurements, and the results are illustrated in Figure 4. Figure 4a shows the full survey spectrum and demonstrates the coexistence of Bi, O, I, and C elements. Specifically, the Bi, O, and I elements resulted from BiOI materials, while the C element may have been derived from the CO_2_ introduced during the test. The spectrum of Bi 4f is shown in Figure 4b. The strong peaks located at 163.98 and 158.68 eV are ascribed to the Bi 4f_5/2_ and 4f_7/2_ spin states of Bi^3+^, respectively [38]. Figure 4c shows the high-resolution spectrum of O 1s, and the main peak at 529.53 eV could be attributed to the Bi-O bond in the [Bi_2_O_2_] layer of the BiOI structure. The secondary peak at 530.93 eV was assigned to the hydroxyl groups of absorbed water on the surface of BiOI [33]. In Figure 4d, two peaks at 630.08 and 618.58 eV resulted from I 3d_3/2_ and I 3d_5/2_ of I^−^, respectively [39].

#### 3.1.4. Optical Properties and Mott–Schottky Analysis

**Figure 5:**
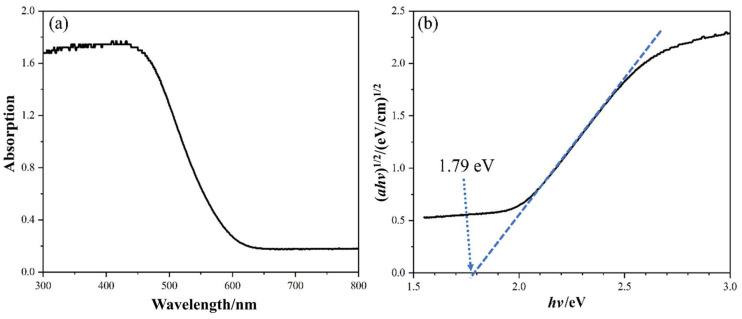
UV-Vis DRS of samples: (**a**) the relationship between absorption and wavelength and (**b**) transformed plot.

The UV-Vis absorption spectrum of BiOI in Figure 5 shows the optical properties. The absorption curve shown in Figure 5a demonstrates that the synthesized BiOI possessed excellent visible light absorption performance. Moreover, the spectrum was transformed using Equation (1) to obtain the band gap of BiOI [40]:

(1) $${\left(\alpha hv\right)}^{\frac{n}{2}}=A\;\left(hv-E_{g}\right)$$

where α is the absorbance index proportional to absorption data, *h* is the Planck constant, *v* is the light frequency, *A* is a constant, and *E_g_* is the band gap. The value of *n* is 1 because BiOI is an indirect semiconductor [31,41]. Figure 5b shows the plots of the (αhv)^1/2^ vs. photon energy, and the 1.79 eV of band gap obtained by the intercept of the *x* axis. Generally, electrons at the valence band (VB) of an indirect semiconductor need to absorb more photon energy than the band gap to leap to the conduction band (CB) because of additional travel along a certain *k*-space distance. On the other hand, the photogenerated carriers of indirect conductors are more difficult to recombine than those of direct conductors [42]. Therefore, the small band gap of BiOI enables the electrons at the top of VB to be efficiently excited to the CB by visible light to form active radicals for photocatalytic reactions. Additionally, the relatively slow recombination rate of photogenerated carriers enhances photocatalytic activity.

**Figure 6:**
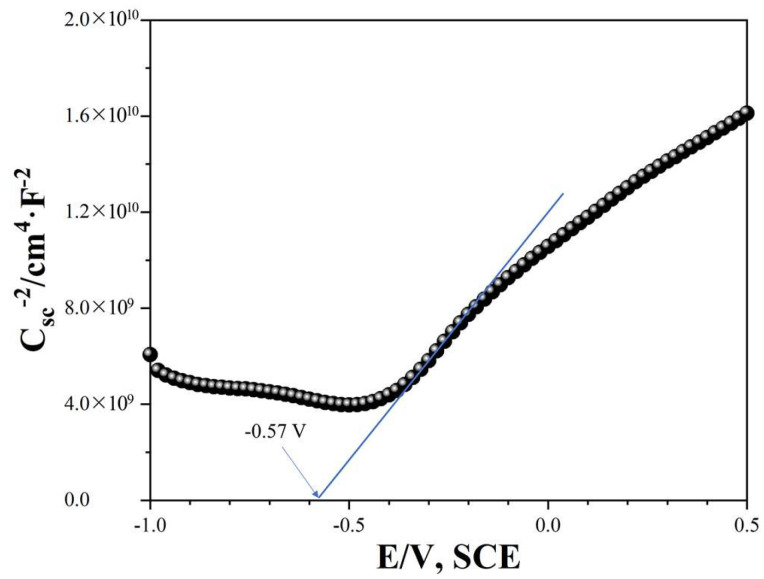
Mott–Schottky plot of BiOI.

Mott–Schottky analysis was carried out to determine the flat band potential (E_f_) and semiconductor type of BiOI. As shown in Figure 6, the positive slope in the Mott–Schottky plot indicates that the synthesized BiOI belonged to an n-type semiconductor. Therefore, the concentration of flowing electrons was greater than that of holes [43]. In addition, the E_f_ obtained by the extrapolation of Mott–Schottky curve was −0.57 V vs. SCE, which corresponds to −0.33 V vs. NHE according to the equation of E_(NHE)_ = E_(SCE)_ + 0.24 V. The E_CB_ of BiOI was estimated to be −0.43 V vs. NHE because of the relationship of E_CB_ = E_f_ − 0.10 V [11]. Combining the value of the band gap, the E_VB_ could be calculated as 1.36 V vs. NHE through E_VB_ = E_CB_ + E_g_. Based on the band structure, the photogenerated electrons on the surface of BiOI had enough capacity to produce ⋅O_2_^−^ (−0.28 V vs. NHE) [37], while ⋅OH could not be generated from OH^−^ (1.99 V vs. NHE) [44] and H_2_O (2.30 V vs. NHE) [45] by photogenerated holes.

### 3.2. Photocatalytic Experiments

#### 3.2.1. Photocatalytic Degradation of Xanthate

**Figure 7:**
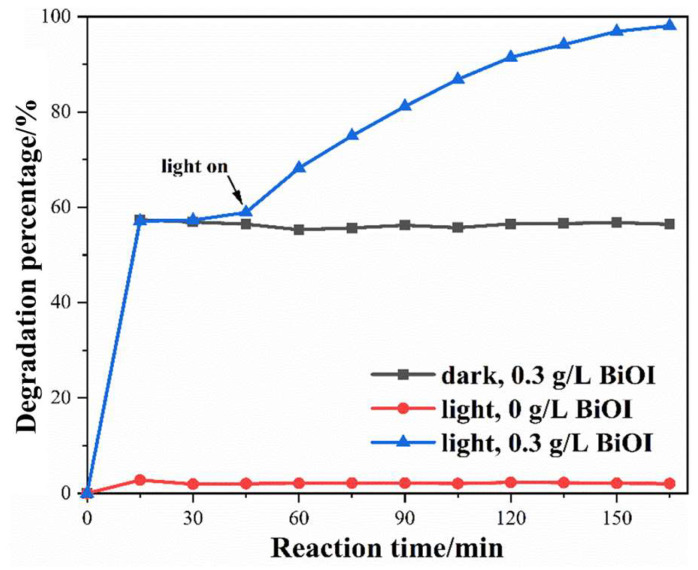
Blank tests of xanthate photodegradation.

Figure 7 shows the results of two blank tests for xanthate photodegradation to determine the self-degradation and adsorption of xanthate. In the absence of BiOI, only 2.28% of xanthate was degraded after 165 min of irradiation, indicating that the self-degradation of xanthate molecules is weak under irradiation of visible light. When xanthate was removed with 0.3 g/L BiOI catalyst in a dark environment, its removal percentage rose rapidly by adsorption in the first 15 min, and then stabilized at 56.50% in the subsequent period. This indicates that xanthate molecules will no longer be removed after their adsorption onto the surface of BiOI reaches equilibrium. In addition, an experiment with both a photocatalyst (0.3 g/L) and a light source was introduced for comparison. During the dark reaction period of the first 45 min, the removal percentage remained at about 56.50%, which is consistent with the adsorption results above. However, the degradation percentage increased rapidly as the light turned on, and gradually climbed to 98.08% after 120 min of irradiation. Thus, both illumination and photocatalysts are necessary for the efficient degradation of xanthate. In addition, the degradation turnover (dTON) [46], which was calculated using (*M_i_* − *M_f_*)/(*t*·cat.), is given to facilitate the comparison of catalysts. In the formula, *M_i_* and *M_f_* are the initial and final amount of the xanthate during degradation, respectively; *t* represents reaction time; and cat. denotes the catalyst amount. Thus, the dTON of BiOI in the degradation of xanthate is 828 μmol·h^−1^·g^−1^.

**Figure 8:**
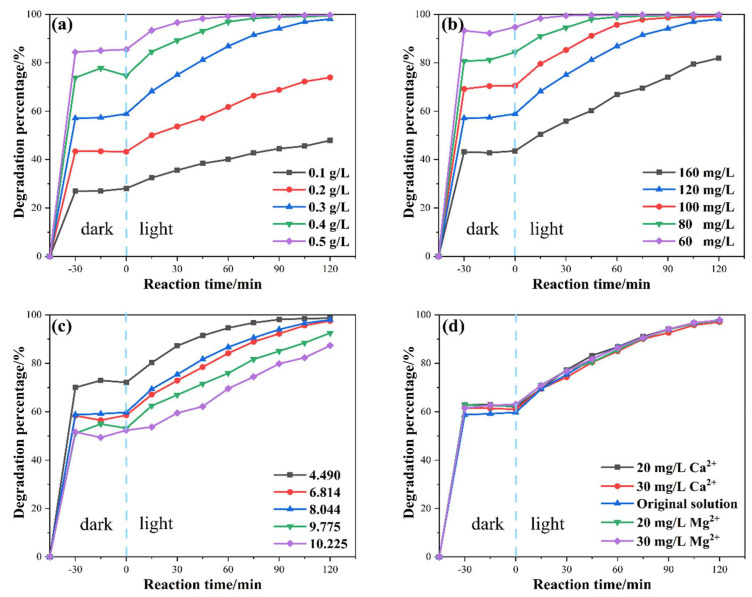
The effects of (**a**) catalyst dosage, (**b**) xanthate concentration, (**c**) pH value of solution, and (**d**) the concentration of calcium and magnesium ions on photocatalytic degradation of xanthate.

The effects of parameters including catalyst dosage, xanthate concentration, pH value of solution, and the concentrations of Ca^2+^ and Mg^2+^ ions on the photocatalytic degradation of xanthate were then studied to evaluate the applicability of BiOI in complex environments. Figure 8a shows the impact of catalyst dosage on the photocatalytic degradation of xanthate at a concentration of 120 mg/L. In the dark adsorption stage, the removal percentage of xanthate increased from 28.06 to 85.49% as the catalyst dosage increased from 0.1 to 0.5 g/L. Moreover, the degradation percentages after 120 min of irradiation at 0.1, 0.2, 0.3, 0.4, and 0.5 g/L conditions reached 47.90, 73.95, 98.08, 99.40, and 99.72%, respectively. Both the increase in dark adsorption and in photodegradation occurred because a larger dosage provides more available specific surface area, in turn providing more active sites for the adsorption and photodegradation of xanthate molecules. Therefore, more photogenerated electrons and holes could be generated and, thus, participate in the degradation reaction. The xanthate degradation achieved 98.08% at the BiOI dose of 0.3 g/L and leveled off after the dose surpassed 0.3 g/L. Therefore, the catalyst dosage for the subsequent degradation experiment was selected as 0.3 g/L.

Generally, the concentration of xanthate in practical applications is 50–150 mg/L [47], so in this section, xanthate concentrations of 60, 80, 100, 120 and 160 mg/L were selected. Figure 8b shows the removal efficiency of xanthate at different xanthate concentrations. The xanthate degradation percentage reached 98.67% and above within 90 min of the photocatalytic reaction when the xanthate concentration was lower than 120 mg/L. Regarding the xanthate concentration of 120 mg/L, the degradation percentage of xanthate was reduced to 94.15% within 90 min of irradiation; it further climbed to 98.08% after 120 min of the photocatalytic reaction. When the xanthate concentration was 160 mg/L, only 81.94% of xanthate was removed within 120 min. In short, photocatalytic efficiency decreases as xanthate concentration increases. This is due to the limited active sites provided by 0.3 g/L BiOI photocatalysts. Xanthate molecules can only be degraded after adsorption onto the active site. Therefore, an overly low xanthate concentration cannot make full use of BiOI photocatalysts, while an excessively high concentration of xanthate cannot be efficiently removed using BiOI. Thus, for subsequent degradation experiments, the xanthate concentration was selected as 120 mg/L.

Figure 8c shows the influence of the pH value of the solution on the photocatalytic degradation of xanthate. As the pH level increased from 4.490 to 10.225, the xanthate adsorption percentage decreased from 72.10 to 52.33% during 45 min of dark reaction, and the xanthate degradation efficiency decreased from 98.70 to 87.33% after a further 120 min of irradiation. The decline was due to the weakening of the electrostatic force between BiOI and xanthate molecules. Specifically, the isoelectric point of BiOI is 2.9; its surface potential became more negative with increasing pH values [48]. As isobutyl sodium xanthate is an anionic collector, it was negatively charged in the solution. Therefore, the electrostatic repulsion between the xanthate molecule and the BiOI nanoparticles became stronger, resulting in the decrease in adsorption and photodegradation. It is worth noting that the pH of xanthate wastewater in actual beneficiation ranges from 10 to 11 [49]. The results show that at a pH value of 10.225, BiOI still has high photocatalytic activity, and the degradation percentage can reach 87.33% after 120 min of irradiation. This indicates that the prepared BiOI photocatalyst can be utilized to remove xanthate from flotation wastewater in real applications.

In practice, Ca^2+^ and Mg^2+^ ions are prevalent in flotation wastewater, and the flotation recovery rate usually decreases with the increase in their concentrations [50]. Therefore, it is of great importance to investigate the effects of Ca^2+^ and Mg^2+^ ions on the photocatalytic degradation of xanthate. Figure 8d presents that the addition of Ca^2+^ or Mg^2+^ has an insignificant influence on the degradation of xanthate, which favors the application of removing residual xanthate from flotation wastewater using BiOI.

**Figure 9:**
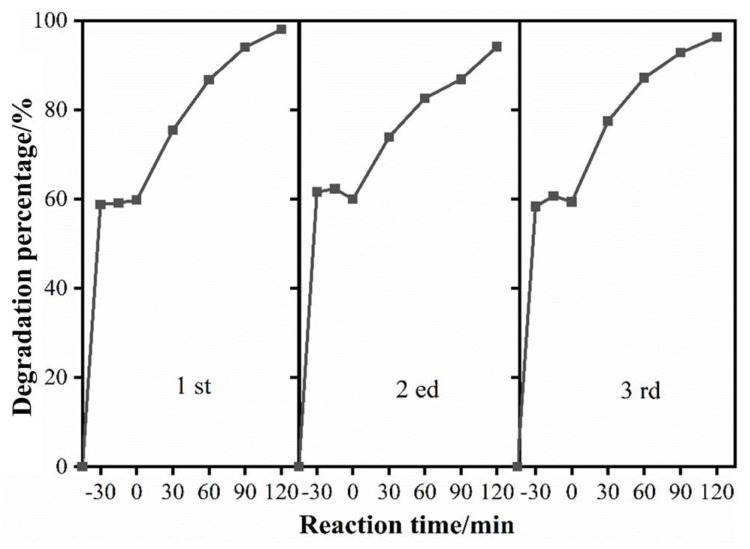
Cycle tests of photodegradation of xanthate. Reaction condition: 120 mg/L of xanthate, 0.3 g/L of catalyst dosage.

Figure 9 shows the cycle tests of photodegradation xanthate by BiOI to explore its stability and reusability. After each photodegradation cycle, the photocatalyst was recovered by centrifugation and then washed with ultrapure water in an ultrasonic bath. The cleaned photocatalysts were then dried at 60 °C for the next cycle. The photocatalytic efficiency hardly changed, and it remained at 96.26% after three cycles. This indicates the good photostability of the as-prepared BiOI.

In summary, the results regarding the photocatalytic degradation of xanthate under different conditions have proven that BiOI possesses good stability and has the capability to treat mineral processing wastewater in real practice.

#### 3.2.2. The Photocatalytic Degradation of NO

**Figure 10:**
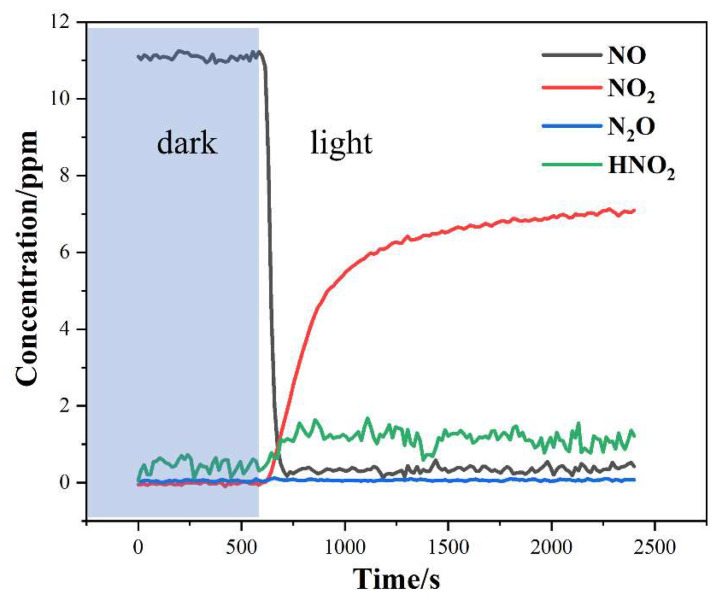
Photocatalytic removal of NO and its sacrificial agent experiment.

Figure 10 shows the concentrations of NO and generated NO_2_, N_2_O, and HNO_2_ over time at the outlet. The concentration of NO stabilized at 11 ppm in the first 10 min under dark conditions, indicating the adsorption equilibrium of NO on the catalyst surface. However, it dropped rapidly once the light was turned on, and finally remained at about 0.35 ppm. Meanwhile, the concentrations of NO_2_ and HNO_2_ in the gas phase increased readily, while that of N_2_O did not change before or after the light was turned on. It can be seen that the summation of NO_2_ and HNO_2_ was less than the initial NO concentration, indicating the formation of NO_x_^−^ ions on the catalyst surface based on the balance of nitrogen elements [51]. Thus, the degraded NO is believed to have been mainly converted into NO_2_ and NO_x_^−^ ions, as well as a small part of HNO_2_. In addition, a minor rise from 0.35 to 0.45 ppm was observed during the 30 min NO removal process. This may be attributed to the accumulation of NO_3_^−^ on the surface of the BiOI [52].

**Figure 11:**
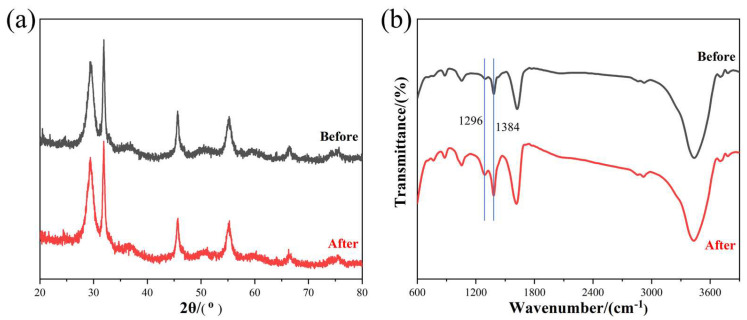
The (**a**) XRD and (**b**) FTIR spectra of BiOI before and after photocatalytic NO removal.

The stability of BiOI was analyzed via the XRD and FTIR spectra. Figure 11a shows the nearly identical XRD patterns of BiOI before and after use, indicating its good and stable crystal structure. The FTIR spectra of BiOI before and after NO removal are shown in Figure 11b. Comparing them, we found that only peaks located at 1296 and 1384 cm^−1^ increased, while the other peaks were almost unchanged before and after photocatalytic NO removal. The unchanged peaks indicate no breaks in chemical bonds. The growing peaks at 1296 and 1384 cm^−1^ should be attributed to the accumulation of NO_2_^−^ and NO_3_^−^ on the catalyst surface, respectively [12,17,53].

Table 1 lists the performances of different catalysts in terms of xanthate and NO removal. For the xanthate, although the degradation efficiency of previous studies could generally reach 90%, the xanthate concentration degraded by the proposed BiOI was the largest (120 mg/L). This indicates that BiOI has good activity in photocatalytic xanthate removal. For the NO, their performances was compared based on light source, gas hourly space velocity (GHSV), initial concentration of NO, and NO removal efficiency. BiOI showed the highest NO removal efficiency among the listed literature works, although it performed at high inlet NO concentrations of 11,000 ppb. In addition, the higher GHSV (480 h^−1^) of this work means a shorter residence time than previous works, requiring strong activity of BiOI for NO removal. In this way, BiOI outperformed its competitors, as shown in Table 1, in photocatalytic NO removal.

In conclusion, the efficient photocatalytic degradation of xanthate (98.08%) from wastewater and the removal of nitric oxide (about 96.36%) from simulated flue gas indicate that BiOI could be used for both wastewater treatment and air pollution control. The reaction mechanisms are elaborated upon below.

### 3.3. Photocatalysis Mechanisms

#### 3.3.1. Main Active Species

**Figure 12:**
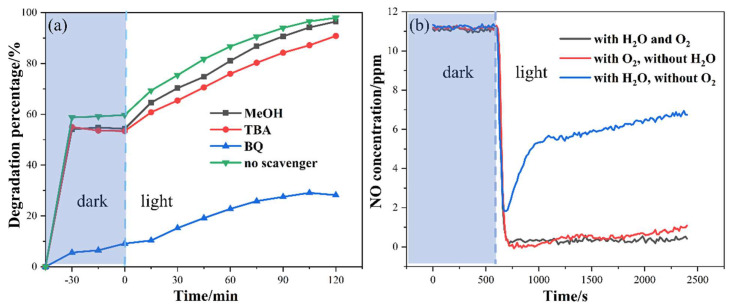
(**a**) Scavenging tests of photocatalytic degradation of xanthate and (**b**) photodegradation of NO without H_2_O or O_2_.

Scavenging tests were carried out to study the role of active species during the photocatalytic reaction. In the photodegradation of xanthate, as mentioned above in the Experimental Section, methanol (MeOH), tert-butanol (TBA), and p-benzoquinone (BQ) were added into 50 mL xanthate wastewater to capture photogenerated electrons (h^+^), hydroxyl radicals (·OH), and superoxide radicals (·O_2_^−^), respectively [35,36]. As can be seen from Figure 12a, the addition of MeOH and TAB had a negligible impact on the photocatalytic degradation of xanthate. This suggests that h^+^ and ·OH radicals play a limited role in xanthate degradation. However, the degradation percentage was reduced drastically from 97.96 to 28.19% when BQ was added. This indicates that the ·O_2_^−^ is the main active specie in the photocatalytic degradation of xanthate.

In the photooxidation of gaseous NO, the added solid scavenger may have covered active sites on the surface of the photocatalyst, thereby affecting its photocatalytic activity. This may be the reason for the contradictory conclusions in previous reports [31,32]. Therefore, in order to avoid unknown surface interactions between scavengers and BiOI, the production of ·OH and ·O_2_^−^ was avoided by removing water and oxygen during the NO photocatalysis, respectively. As shown in Figure 12b, the NO removal efficiency without H_2_O dropped slightly compared to that at RH of 50–70%, which shows that ·OH radicals were not the main active species. Conversely, the photocatalytic activity of BiOI was obviously inhibited when O_2_ was absent, indicating the main role of ·O_2_^−^ in NO photodegradation.

According to the band structure analyzed in Section 3.1.4, the photogenerated electrons on the surface of BiOI have enough capacity to produce ·O_2_^−^ (−0.28 V vs. NHE) [37], while ·OH cannot be generated from OH^−^ (1.99 V vs. NHE) [44] and H_2_O (2.30 V vs. NHE) [45] by photogenerated holes. However, the ⋅O_2_^−^ could react with H_2_O to generate ⋅OH, which could also participate in the degradation reaction [62]. This theoretically explains why the ·O_2_^−^ radicals are primary active species, and ⋅OH radicals play a secondary role in the photodegradation of both NO and xanthate using BiOI. Such a conclusion is consistent with the results of scavenging tests.

#### 3.3.2. Preliminary Exploration of Degradation Process

**Figure 13:**
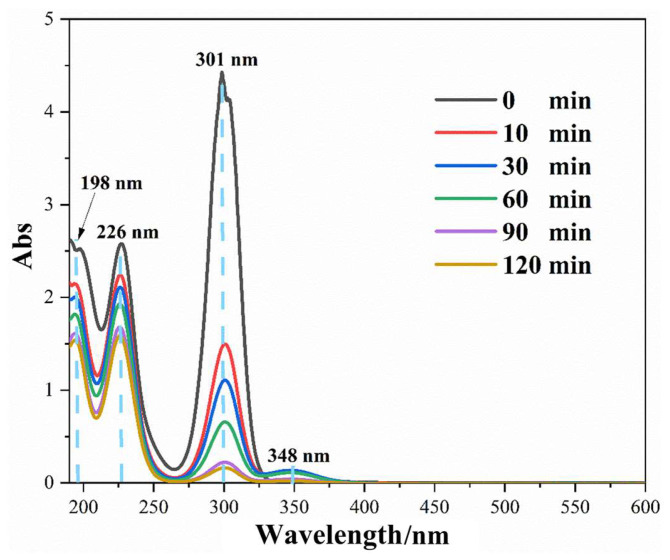
Full-spectrum scanning for the degradation process of xanthate.

The samples collected during the photodegradation of xanthate were scanned using a UV-Vis spectrophotometer ranging from 190 to 550 nm to determine intermediate products. As shown in Figure 13, the absorption peak at 301 nm [49], which was the characteristic absorption peak of xanthate, was reduced significantly as the reaction proceeded. Moreover, the second absorption peak of xanthate at 226 nm also decreased with time, showing the breakdown of xanthate molecules. Prior to irradiation, there was no absorption peak at 348 nm [49] (characteristic peak of peroxide xanthate), while a tiny peak at this position appeared immediately once the light was turned on. However, this tiny peak disappeared at the end of the photocatalytic reaction, indicating that peroxide xanthate is only an intermediate. The absorption peak at 198 nm was assigned to solvent [63]. In addition, COD was measured to study the mineralization degree of xanthate after photodegradation. In total, 71.01% of xanthate was mineralized into inorganic salts, which is close to the COD value of photodegradation of xanthate using Bi_2_WO_6_ [34].

In summary, peroxide xanthate is an intermediate generated during the photocatalytic degradation of xanthate using BiOI. Such a process does not completely convert xanthate into inorganic products, and the final products contain some small-molecule organics. The specific degradation process remains to be further studied in the future. 

For the photocatalytic degradation of NO, the products mainly include NO_2_, NO_3_^−^, and HNO_2_, according to the results in Section 3.3.2. Combined with the main active species, the reaction route of NO is proposed as follows:

BiOI + *hv* → *e*^−^ + *h^+^* (2)

*e*^−^ + O_2_ →·O_2_^−^ (3)

·O_2_^−^ + 2H_2_O → 4 ·OH + *e*^−^ (4)

2 NO + ·O_2_^−^ → 2 NO_2_ + *e*^−^ (5)

NO + 2 ·OH → NO_2_ + H_2_O (6)

NO + ·OH → HNO_2_ (7)

NO + 3 ·OH → HNO_3_ + H_2_O (8)

NO_2_ + ·OH → HNO_3_ (9)

NO + ·O_2_^−^ → NO_3_^−^ (10)

NO_2_^−^ + 2 ·OH → NO_3_^−^ + H_2_O (11)

First, the electrons on VB are excited to CB by visible lights, generating photogenerated *e*^−^ and *h^+^* as demonstrated in Equation (2). Then the *e*^−^ reacts with O_2_ to form ·O_2_^−^ groups, and partial ·O_2_^−^ radicals further combine with H_2_O to produce few ·OH radicals, as shown in Equations (3) and (4). Finally, these active species convert NO into products, including NO_2_, HNO_2_, and HNO_3_, following Equations (5)–(11).

## 4. Conclusions

The following conclusions can be drawn from this study.

(1) BiOI microspheres prepared using the solvothermal method were used to degrade residual xanthate and gaseous NO. The results show that a xanthate degradation of 98.08% can be achieved at an initial xanthate concentration of 120 mg/L with 0.3 g/L of photocatalysts. Furthermore, about 96.36% of NO removal efficiency can be accomplished at a NO inlet concentration of 11 ppm, 5% O_2_, and 50–70% RH.
(2) The effects of parameters including photocatalyst dosage, xanthate concentration, pH value, and concentrations of Ca^2+^ and Mg^2+^ ions on the removal of xanthate were investigated. As the dosage increased from 0.1 to 0.5 g/L, the xanthate degradation increased from 47.90 to 99.72%. As the xanthate concentration increased from 60 to 160 mg/L, the xanthate degradation dropped from 99.86 to 81.94%. Furthermore, acidic conditions are conducive to the photodegradation of xanthate, while alkaline conditions inhibit degradation. Photocatalytic degradation of xanthate can achieve an efficiency of 87.33% under a pH value of 10.225 (which is close to the pH level of flotation wastewater). Moreover, calcium and magnesium ions have an insignificant impact on xanthate photodegradation.
(3) The reaction path and mechanism of photodegradation of xanthate were discussed based on scavenging tests, chemical oxygen demand (COD) measurements, and full-spectrum scanning. In the photocatalytic degradation of xanthate molecules, ·O_2_^−^ radicals were the main active species and resulted in a mineralization degree of 71.01%. Furthermore, the xanthate degradation dropped slightly from 98.08 to 96.26% after three cyclic tests, indicating that the as-synthesized BiOI photocatalysts had good stability and high potential for engineering applications.
(4) Finally, degraded NO is believed to be mainly converted into NO_2_ and NO_x_^−^ ions, as well as a small part of HNO_2_, during NO photocatalysis, and the main active species are also the ·O_2_^−^ radicals.

## Figures and Tables

**Table 1:** The xanthate and NO removal performances of different materials.

Photocatalyst for Xanthate	Light Source	Concentration of Xanthate (mg/L)	Reaction Time (min)	Degradation Efficiency (%)	Ref.
Ag-TiO_2_-FAMB	visible light	10	1800	98.50	[54]
CuO/g–C_3_N_4_	visible light	50	120	83.20	[55]
PANI/TiO_e_/metakaolin	visible light	100	240	94.80	[56]
TiO_2_/graphene	simulated sunlight	20	130	97.03	[57]
TiO_2_/g-C_3_N_4_	simulated sunlight	20	130	97.10	[49]
BiOI	visible light	120	175	98.08	This work
**Photocatalyst for NO**	**Light Source**	**GHSV (h^−1^)**	**Concentration of NO (ppb)**	**Degradation Efficiency (%)**	**Ref.**
PI–g-C_3_N_4_	simulated sunlight	53.10	600	66.00	[58]
Pd-Cv-g-C_3_N_4_	simulated sunlight	22.67	2200	56.30	[59]
Co_3_O_4_/g-C_3_N_4_	simulated sunlight	31.86	600	57.00	[60]
BiOBr/SnO_2_	visible light	32.00	600	50.30	[61]
BiOI	visible light	480	11,000	96.36	This work

## Data Availability

Data are contained within the article.
